# Increasing utilisation of perinatal services: estimating the impact of community health worker program in Neno, Malawi

**DOI:** 10.1186/s12884-019-2714-8

**Published:** 2020-01-06

**Authors:** Chiyembekezo Kachimanga, Elizabeth L. Dunbar, Samuel Watson, Katie Cundale, Henry Makungwa, Emily B. Wroe, Charles Malindi, Lawrence Nazimera, Daniel Palazuelos, Jeanel Drake, Thomas Gates, Thomas van den Akker, Jawaya Shea

**Affiliations:** 1 Partners In Health, Neno, Malawi; 2 Department of Peadiatrics and Child Health, Faculty of Health Sciences, University of Cape Town, Cape Town, Republic of South Africa; 3 Partners In Heath, Harper, Liberia; 4 Warwick Medical School, University of Warwick, Coventry, UK; 5 Ministry of Health, Neno, Malawi; 6 Partners In Health, Boston, USA; 7 FHI 360, Washington, DC USA; 8 Independent, Lancaster, PA USA; 9 Department of Obstetrics, Leiden University Medical Center, Leiden, Netherlands; 10 Athena Institute, VU University, Amsterdam, the Netherlands

**Keywords:** Maternal mortality, Perinatal care, Antenatal care, Deliveries, obstetric, Postnatal care, Intrapartum care, Malawi, Synthetic control, quasi-experimental study, Community health workers

## Abstract

**Background:**

By 2015, Malawi had not achieved Millennium Development Goal 4, reducing maternal mortality by about 35% from 675 to 439 deaths per 100,000 livebirths. Hypothesised reasons included low uptake of antenatal care (ANC), intrapartum care, and postnatal care. Involving community health workers (CHWs) in identification of pregnant women and linking them to perinatal services is a key strategy to reinforce uptake of perinatal care in Neno, Malawi. We evaluated changes in uptake after deployment of CHWs between March 2014 and June 2016.

**Methods:**

A CHW intervention was implemented in Neno District, Malawi in a designated catchment area of about 3100 women of childbearing age. The pre-intervention period was March 2014 to February 2015, and the post-intervention period was March 2015 to June 2016. A 5-day maternal health training package was delivered to 211 paid and supervised CHWs. CHWs were deployed to identify pregnant women and escort them to perinatal care visits. A synthetic control method, in which a “counterfactual site” was created from six available control facilities in Neno District, was used to evaluate the intervention. Outcomes of interest included uptake of first-time ANC, ANC within the first trimester, four or more ANC visits, intrapartum care, and postnatal care follow-up.

**Results:**

Women enrolled in ANC increased by 18% (95% Credible Interval (CrI): 8, 29%) from an average of 83 to 98 per month, the proportion of pregnant women starting ANC in the first trimester increased by 200% (95% CrI: 162, 234%) from 10 to 29% per month, the proportion of women completing four or more ANC visits increased by 37% (95% CrI: 31, 43%) from 28 to 39%, and monthly utilisation of intrapartum care increased by 20% (95% CrI: 13, 28%) from 85 to 102 women per month. There was little evidence that the CHW intervention changed utilisation of postnatal care (− 37, 95% CrI: − 224, 170%).

**Conclusions:**

In a rural district in Malawi, uptake of ANC and intrapartum care increased considerably following an intervention using CHWs to identify pregnant women and link them to care.

## Introduction

Malawi’s maternal deaths decreased by approximately 35%—from 675 to 439 deaths per 100,000 live births—between 2010 and 2016 [1–3]. This fell short of the Millennium Development Goal (MDG) target of 150 deaths per 100,000 live births [2, 4]. The Sustainable Development Goals (SDGs) introduced a new target of reducing the maternal mortality ratio (MMR) even further to 70 per 100,000 live births by 2030. This requires strengthening current best practices in Malawi, and developing new strategies for reducing maternal deaths [4].

In Malawi, up to one third of maternal deaths occur during the antenatal period [5]. Women who die during pregnancy are less likely to attend any ANC visit and often attend less than the four recommended ANC visits [5]. Moreover, significant maternal deaths occur outside health facilities, with one study showing 38% of maternal deaths happening outside the health facilities [6]. In addition, as many as half of maternal deaths are attributed to delays in seeking perinatal care at health facilities [5].

Women experience challenges in utilising perinatal care services in Malawi. In 2015–2016, up to 70% of women of childbearing age experienced at least one or more challenges in utilising perinatal care services. Along the perinatal care cascade, 25% of pregnant women started ANC in the first trimester and only half finished the recommended four or more ANC visits. Although nine of ten pregnant women received intrapartum care at health facilities, about half did not make any postnatal care visits within 6 weeks after facility births [2].

Delays in utilising perinatal services have been attributed to women’s failure to recognise danger signs or adhere to timely attendance of perinatal services, the use of traditional birth attendants for perinatal services, and lack of transport to health facilities [2, 5, 7]. Addressing these challenges ensures women are identified and referred in timely manner to health facilities and may help increase the utilisation of perinatal services and reduce maternal deaths.

Home visits by community health workers (CHWs) could be a valuable strategy for improving utilisation of perinatal care, as CHWs could disseminate heath information, identify pregnant women, and link women to perinatal health services [8, 9]. In Malawi, the impact of CHWs on improving perinatal service utilisation differs between studies. For example, a study of paid maternal-health trained CHWs was implemented in 14 health facilities between 2007 and 2010. The CHWs conducted initial home visits to identify pregnant women, followed by three ANC and three postnatal care home visits. Although the study showed an increase in the proportion of women utilising at least one ANC visit and intrapartum care, it lacked a comparison site, and did not explore the impact of CHWs on the timing and frequency of ANC and postnatal care attendance [10]. A randomised control trial conducted in 13 facilities in one district between 2005 and 2009 did not find evidence to support the role of CHW home visits on ANC attendance, completion of four or more visits, utilisation of intrapartum care, or postnatal care attendance [11, 12]. In this study salaried CHWs received initial maternal health training and conducted up to five home visits during pregnancy and the postnatal period to provide education and refer women to care [12]. With minimal evidence surrounding the impact of CHWs on improving utilisation of perinatal care in Malawi, it is crucial that more research regarding the impact of CHWs on the entirety of the perinatal care continuum, including the timing and frequency of ANC visits, intrapartum care, and postnatal care be conducted.

Prior to 2015, home visits by CHWs were used to improve utilisation of HIV, tuberculosis, Kaposi Sarcoma and palliative care services in Neno District, Malawi [13–15]. In 2015, Neno had the best HIV outcomes in Malawi, with the success partially attributed to the CHW program [14]. Based on gaps within other CHW programs in Malawi and our prior experience in Neno in HIV, tuberculosis, Kaposi Sarcoma and palliative care services, we hypothesised that a CHW program may increase utilisation of perinatal services. Therefore, we implemented a maternal health CHW program aimed at increasing utilisation of maternal health services in Neno. This paper describes our findings upon evaluating a comprehensive CHW program called ‘*Healthy Mothers*, *Healthy Communities’* between 2014 and 2016. The CHW primary roles were to conduct home visits and to facilitate referrals to perinatal services in order to increase utilisation of perinatal services. We estimated the effect of CHWs on uptake of maternal health services; specifically, on the frequency and timing of ANC, intrapartum care, and postnatal care service uptake.

## Methods

### Study setting and design

This study was completed in Neno District, Malawi. Neno—a district in the southern region with an estimated population of 150,000 in 2015—is a rural area with 65% of the people classified as poor in 2011, 15% higher than Malawi’s national average [16, 17]. In 2015, Neno had 11 health centers and two hospitals offering primary and secondary medical services, respectively. CHWs started working in Neno in 2007 and were primarily assigned to HIV and tuberculosis patients to provide education, accompaniment to medical appointments, psychosocial support, and linkage to formal health services [18]. In 2015, approximately 800 CHWs worked in Neno. The CHWs did not have any formal responsibilities towards women requiring ANC, intrapartum care, or postnatal care.

This quasi-experimental study was conducted between March 2014 and June 2016. The study period totalled 28 months, with a pre-intervention phase of 12 months (March 2014 to February 2015), followed by 16 months of a maternal health focused CHW intervention (March 2015 to June 2016). The initial 3 months of the intervention (March to May 2015) included a training period followed by immediate deployment of all CHWs to their catchment areas.

### Intervention description

The *Healthy Mothers, Heathy Communities* program was implemented in two catchment areas: Chifunga and Lisungwi. The catchment areas contained a total of 20,000 people and included an estimated 3100 women of child-bearing age, based on a CHW census conducted during the intervention period. In an effort to equitably distribute CHWs and allow combined HIV, tuberculosis and maternal health activities, CHWs were switched from a patient-based approach in which CHWs were assigned to a specific group of patients, to a household-based approach in which CHWs were assigned to cover a given number of households. Switching to this household approach also had a potential effect of decreasing stigma since CHWs were no longer solely visiting households with HIV and/or tuberculosis patients, but were visiting all households regardless of whether those households had patients. One CHW covered an average of 20 households. The shift to households resulted into changes to the number of CHWs required to cover the catchment areas. As a result, 83 new CHWs were added to the existing cadre of 108 CHWs, bringing the total number of CHWs to 211 in the two catchment areas.

Newly-appointed CHWs were already residing in the assigned catchment area and were identified by the community based on pre-defined criteria (See Table 1A). Key criteria for selecting CHWs included people who lived in the same area where they would work, people with good behaviour who were seen as role models, and those that had previously assisted in community maternal and neonatal initiatives. All CHWs received 1 week of training with specific emphasis on monthly home visits, education, and referral to facilities in addition to their HIV and tuberculosis work. The one-week training included the roles of CHWs, the program’s supervision structure, and key maternal health topics including ANC, intrapartum care, postnatal care, HIV and prevention of mother to child transmission, and management of danger signs during pregnancy. The training also emphasised the process of accompaniment: identifying pregnant women using screening questions in their homes and then physically escorting women to a health facility for pregnancy confirmation as well as the first ANC visit, all subsequent ANC visits, visits to a facility for intrapartum care, two visits in the postnatal period. Accompaniment also included providing psychosocial support to the women; the CHWs were willing to share the ‘journey’ of medical care with their patients during the perinatal period. This process included accompanying patient to routine visits as well as during emergency visits. Accompaniment has been one of the key factors to success of many CHWs in more than 10 countries of the world [19].

**Table 1:** Selection criteria and responsibilities of CHWs

A	B
CHW selection criteria	Responsibilities of CHWs
CHW would be • female; some exceptions will be made for men who fulfil the rest of the criteria • between 25 to 49 years of age; some exceptions will be made for those below 60 years of age who are capable of escorting community members to the health facility • dedicated and very committed to their work • able to read and write • honest and truthful • living within the same village or community • willing to work as a volunteer • well-behaved, accepted and trusted by the community • trusted to keep patient information in confidence at all times • non-drinker and non-smoker • clean at all times • role model in the village • Priority will be given to women who are a part of the maternal and child health support groups	• Conduct monthly visits to all assigned households to identify pregnant women as early as possible, educate women about the importance of starting ANC, tuberculosis,sexually transmitted infection, HIV, family planning, and malnutrition • Escort pregnant women and new mothers to the health facility for initial and all ANC visits, labour and delivery, two postnatal care visits and all emergency visits • During monthly household visits, follow-up with pregnant women and their partners to provide education, making of birth plans and screening for malnutrition • Conduct follow-up visits with mothers and their newborns to Educate about exclusive breastfeeding, Post care visits, and FP options and monitor danger signs in both mother and their infants • Visit HIV and TB patients daily to ensure patient adherence and monitor for danger signs • Visit pre-Antiretroviral patients weekly to ensure they are adhering to their medication and provide education • Escort Tuberculosis and HIV patients for all scheduled appointments and emergency visits.

A) CHW selection criteria for *healthy mothers, healthy communities* project in Neno, Malawi B) CHW responsibilities in their assigned catchment areas

*CHW* Community health workers

After training, CHWs were immediately deployed to communities with the following responsibilities: 1) provide community education; 2) conduct monthly home visits to identify pregnant mothers; 3) escort women to facilities through ANC, intrapartum care, and postnatal care; and 4) record and report monthly activities (Table 1B). The CHWs were not providing medical care during the home visits. The CHWs received a monthly stipend equivalent to US$20 in 2015 and were supervised by a cadre of senior CHWs, at a ratio of one senior CHW to 10 CHWs, as well as one facility-based supervisor at each of the two facilities. Information on home visits was recorded in specially designed registers which were aggregated monthly. Aggregate CHW activities was shared with the health facilities every month.

### Other supporting interventions

To ensure that facilities were able to cope with any increased demand, a maternity waiting home was constructed at each of the CHW intervention facilities. Additionally, the facilities received needed medical supplies to ensure they were able to provide a minimum package of care in ANC, intrapartum care, and postnatal care. Due to an acute shortage of nursing staff, one additional nurse was hired at Chifunga Health Center. At the request of the communities, 23 bicycle ambulances were provided to the catchment areas to help ease transport from the community to the facility during obstetric emergencies. Two mass awareness campaigns per quarter were conducted to update the community on the progress of the CHW intervention.

### Selection of intervention and control sites

The two facilities where the maternal health CHW program was implemented (Chifunga and Lisungwi) were chosen based on Ministry of Health ownership and their ability to provide ANC, intrapartum care, and postnatal care services. In Malawi, services in public health facilities are free at the point of use. Chifunga Health Center is a primary health facility offering basic emergency obstetric services, while Lisungwi Community Hospital is a secondary health facility offering comprehensive emergency obstetric care.

The control areas were also primarily based on Ministry of Health ownership within Neno District and the facility’s ability to provide one or more of the perinatal services of interest in this study. Based on these criteria, six health facilities were eligible for inclusion in the study as potential control sites (Additional file 1).

### Data collection

The sample comprised of the two intervention sites and six control sites. Analysis was conducted at the cluster level as only aggregate data were available. The cluster-level outcomes were derived from all women of childbearing age who utilised perinatal services. Data were obtained from ANC, maternity, and health management information systems reports. This included routine reports aggregated and produced by the facility and sent to the district health information system (DHIS II) every month from March 2014 to June 2016. The monthly report consisted of aggregated and anonymised data with no individual patient-level data. Where data for specific months were missing, it was requested from the reporting facilities and if still missing, the facilities were asked to rewrite the report from the source register. In instances where the source register was not found, data was removed from analysis. Formal data quality assessment was not done. Due to nature of the data, the need for informed consent was waived.

The following outcomes used in this study were observed monthly at the cluster level: 1) the number of new women enrolled in ANC care; 2) the proportion of enrolled women starting ANC in the first trimester; 3) the proportion of enrolled women completing four or more ANC visits; 4) the monthly number of women utilising intrapartum care at the health facility; and 4) the monthly number of women receiving postnatal care within 2 weeks after facility births.

All monthly data before and after the CHW intervention was initially extracted in Microsoft Excel 2013 and then aggregated and cleaned using Stata 14.2. Summary statistics were calculated in Stata 14.2 and further statistical analysis was conducted using R 3.4.1 software.

### Statistical methods

The two intervention sites, Chifunga and Lisungwi catchment areas, were treated as one ‘treatment’ area for the purposes of analysis, as they are one contiguous area in which the intervention was implemented at the same time. Multiple possible control sites were available to estimate the causal effects of the intervention on the outcomes of interest. Rather than making a choice of one particular control site, a “synthetic control” method was used to estimate an artificial (or synthetic) counterfactual site that replicates the outcomes that would have been observed in the intervention site had the intervention not been implemented [20, 21]. The counterfactual site outcomes are estimated by taking a weighted average of the control site outcomes so that they replicate the intervention site outcomes in the pre-intervention period. These estimated weights are then applied in the post-intervention period to generate the counterfactual time series against which the treatment site outcomes can be compared, and treatment effects estimated. These methods have been applied in other health service research settings [22–26] .

The approach to the synthetic control method used in this study was proposed by Brodersen et al. who developed a Bayesian formulation on the basis of a general state-space model [22]. These models also permit other control time series and dynamic effects of covariates, however no other covariates were used in the model for this study. To select among the control sites—i.e. generate the weights to predict the counterfactual outcome—a ‘slab and spike’ prior was used. The posterior predictive distribution of the counterfactual time series is generated from which treatment effects and credible intervals are determined. Average treatment effects are estimated by averaging over the whole post-intervention period, but counterfactual time series are reported for each month as well. The R package *CausalImpact* was used to estimate the models [22].

## Results

### Summary statistics

During the first 16 months of implementation, 1563 new women were enrolled into ANC care in the CHW intervention site. Utilisation of ANC, intrapartum care, and postnatal care increased in comparison to the 12 months prior to the implementation of the CHW program (Table 2). The proportion of women starting ANC in the first trimester increased from 13% (10 women out of 79 women per month) to 29% (29 women out of 103 per month), women completing four or more ANC visits increased from 28% (22 women per month) to 39% (40 women per month), intrapartum care increased from 85 births to 102 births per month, and women attending postnatal care increased from 44 women to 50 women every month. Except for the utilisation of postnatal care, control sites also exhibited increases between pre- and post-intervention, but increases were minimal in comparison to the CHW intervention sites (Table 1).

**Table 2:** Summary outcomes in CHW Intervention sites and control sites

Facility	New ANC		% ANC in first trimester		% Four or more ANC		Facility births^c^		PNC	
	Pre-intervention^a^	Post intervention^b^	Pre-intervention	Post intervention	Pre-intervention	Post intervention	Pre-intervention	Post intervention	Pre-intervention	Post intervention
Chifunga	23.7 (5.4)	39 (12.6)	10.2 (7.9)	37.5 (11.9)	32.5 (21.2)	50.3 (9.4)	20 (4.8)	23.4 (5.5)	11.7 (7.6)	15.7 (10.4)
Lisungwi	55.7 (14.5)	58.7 (13.3)	14.4 (7.1)	23.1 (9.1)	26.5 (9.1)	32.7 (6.6)	65.3 (14.3)	79 (13.2)	32.3 (18.7)	34.4 (24.7)
CHW intervention	79.4 (14.9)	97.7 (23.1)	13.1 (5.0)	28.6 (7.5)	28.5 (3.9)	39.0 (4.7)	85.3 (14.0)	102.4 (13.1)	44 (21.3)	50.1 (28.8)
Ligowe	26.8 (11.2)	34 (7.9)	8.7 (6.1)	24.3 (14.2)^d^	29.2 (15.3)	31.1 (11.8)	–	–	0 (0)	10 (11.8)
Luwani	11.6 (3.8)	14.3 (6.0)	24.3 (15.4)	25.1 (23.4)	33.7 (15.1)	40.5 (14.8)	4.1 (3.4)	2.9 (2.8)	5.8 (2.9)	4.7 (3.0)
Magaleta	35.0 (8.6)	31 (7.5)	4.1 (4.2)	9.2 (7.1)	42.3 (10.4)	55.1 (7.6)	27.4 (6.4)	26.4 (7.0)	17.6 (6.0)	17.5 (11.4)
Neno district hospital	89.9 (23.2)	85.5 (15.2)	15.4 (5.7)	19.2 (5.8)	21.3 (7.6)	21.5 (5.1)	117 (18.6)	125.4 (29.4)	28.7 (15.6)	45.4 (29.4)
Zalewa	33.7 (6.2)	37.7 (6.2)	13.9 (5.2)	19.4 (9.5)	44.3 (9.9)	46.1 (5.4)	–	–	2.2 (1.7)	7.5 (4.1)
Midzemba	–	–	–	–	–	–	–	–	–	–
Control sites	197 (23.4)	202.5 (17.2)	12.5 (2.8)	18.9 (5.0)	30.6 (4.7)	34.0 (3.0)	148.5 (19.5)	154.7 (27.4)	54.3 (14.1)	85.1 (39.1)

Summary statistics of outcomes in intervention and synthetic units.

NB

Values are mean (SD) unless otherwise stated.

% Percentage.

^a^Pre-intervention: March 2014 to February 2015(12 months).

^b^Post-intervention period March 2015 to June 2016(16 months).

^c^Two facilities had no capacity to provide intrapartum care during the study period.

^d^Averaged over 15 months as data for one month was missing in the post-intervention period. Midzemba did not report any of the outcomes of interest during the study period.

### Synthetic control analysis

**Fig. 1:**
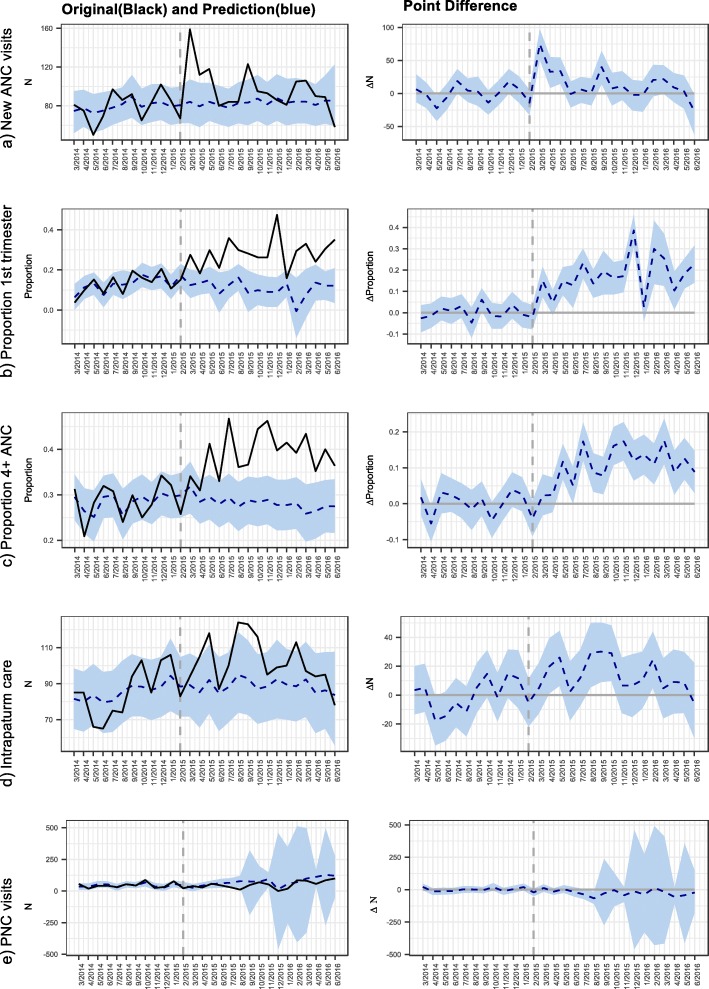
**a**-**e** Synthetic control analysis of CHW impact on perinatal use. The left column shows the intervention cluster outcome time series (solid line) and synthetic control time series (dashed line). The right column shows the difference between the treatment and synthetic control sites. 95% Credible interval is shown by the blue band. The synthetic control method was able to create a pre-intervention CHW time series that was similar with the counterfactual time series with CHW intervention time series lying within the 95% credible region of the counterfactual series. With the exception of Figure 1e, Figure 1(a-d) show an increase in CHW time series after intervention away from the counterfactual time series and its credible region, signifying increases in these outcomes after CHW implementation

Table 3 and Fig. 1a-e summarise the results from the synthetic control analysis. The CHW intervention was associated with a significant increase in the number of new women enrolled in ANC by 18% (95% credible interval (CrI): 8, 29) from 83 women per month in the counterfactual unit to 98 women per month in the CHW intervention. The CHW intervention appeared to have an immediate effect on the number of new women enrolled in ANC—i.e. within the first month—with the highest number of women enrolled within the first 4 months of the intervention (Fig. 1a). After the immediate effect, the new women enrolled in ANC fluctuated from month to month, but was higher than the pre-intervention monthly enrolments. The intervention was also associated with an increase in the proportion of women attending ANC in the first trimester of 200% (CrI: 162,234) from 10 to 29%, and with an increase in the proportion of women completing four or more ANC visits of 37% (CrI: 31, 43) from 29 to 39% (Table 3).

**Table 3:** Results of main outcomes after CHW intervention

Outcome (average per month)	Posterior mean synthetic unit outcome	CHW intervention outcome	Percentage change (%^a^, CrI^b^)
Number of new women enrolled in ANC	83	98	18 (8,29)
% of women starting ANC in first trimester	10	29	200 (162, 234)
% of women completing 4 or more ANC visits	29	39	37% (31,43)
Number of monthly facility births	85	102	20 (13, 28)
Postnatal care visits	79	50	−37 (224, 170)

Main outcomes of CHW intervention between synthetic unit and its control.

^a^ Percentage

^b^ Credible Interval

The CHW intervention was also associated with an increase in the number of women utilising intrapartum care at a facility. Without the intervention, the number of women utilising intrapartum care was estimated to have been an average of 85 per month. With the CHW intervention, the number increased by 20% (CrI: 13, 28) to 102 facility births every month.

There was little evidence to indicate if the intervention influenced the number of women attending postnatal care clinics. While the average number of women attending postnatal care was estimated to decrease by 37%, this was estimated very imprecisely with a 95% credible interval of − 224 to 170%.

## Discussion

The results of this study provide strong evidence to suggest that a maternal health focused CHW intervention can increase the utilisation of ANC and intrapartum care. The effect of the CHW intervention on increasing new women enrolled in ANC was immediately significant. CHWs located many pregnant women who were immediately escorted to the health facility for enrolment in ANC before finding new pregnancies.

The significant effect of the CHW intervention was on improving ANC attendance within the first trimester (200% increase), as recommended by the recent 2016 World Health Organisation ANC guidelines [27]. This intervention managed to triple the proportion of women starting ANC in the first trimester; however, this was from a relatively low baseline of 10%, which leaves plenty of additional room for improvement. The previously low uptake reflects the national trend in Malawi, and in fact the national target for first trimester ANC visits during this study period was just 20% [28]. The low uptake of ANC in the first trimester has been attributed to fear of witchcraft, embarrassment if pregnancy is not carried to term, and gossip [29]. In this study, CHW-mediated support including household education support, emotional support, consistent reminders to women to attend facilities for care, and escorting the women to care contributed to the increased ANC utilisation.

The 2015/2016 Demographic and Health Survey showed that nine in ten women in Malawi obtain intrapartum care at a health facility [2]. In a program aimed at increasing facility births, the level of improvement is minimal if almost all women have intrapartum care within a facility. Results from studies in Malawi and other countries have not shown a significant effect of CHW programs on improving facility-based births [10, 29–31]. However, this study shows that it was possible to increase the number of women giving birth in a health facility in Malawi by using maternal health focused CHWs.

This study did not find evidence of an improvement in utilisation of postnatal care after the CHW intervention, although the estimates had a high degree of uncertainty. Studies of the effect of CHW programs on postnatal care report mixed results. For example, a study in Bangladesh showed an increase in postnatal care utilisation, while two studies in Ethiopia showed little to no effect of various CHW programs on postnatal care uptake [32–34]. It will be important to investigate whether the intervention impacted postnatal care use in Neno and if not, why this was the case. One reason that may contribute to unchanged postnatal care visits in Neno may be because of the poor quality of postnatal care data in Neno. As compared to ANC and monthly facility births data, postnatal care clinics do not have well-defined registers and specific monthly reporting tools. Additionally, postnatal care receives minimal supervision from district level supervisors compared to ANC and intrapartum care. It is therefore possible that women were attending postnatal care clinics but were not recorded in the proper postnatal care registers.

This study has some weaknesses. The synthetic control method provides a way of estimating causal effects from observational data that is stronger than other study designs, such as before and after studies or difference in difference analyses with a single control site. However, this method still relies on (untestable) assumptions that may fail in practice. For example, these include an assumption of no spill-over between intervention and control sites, and that the relationship between control sites and intervention site remains stable. Nevertheless, these biases would be unlikely to affect our conclusions, which conform to our prior expectations of the intervention, and are likely to be small in magnitude relative to the effect of the intervention which is substantial.

The CHW intervention and related facility activities may have strengthened service provision, data recording, and reporting in the CHW intervention sites. Changes in outcomes could also be related to improved reporting rather than a change in the underlying outcome itself. Additionally, as the CHW intervention was implemented, some improvements at the health facilities were carried out to ensure women were able to obtain quality care. This may also have increased the number of women utilising the services as they perceived better quality of care within the facilities. Finally, it would have been important measure the cost of the program as this may impact its feasibility in other settings.

This study measured changes in utilization of ANC, facility based births and postnatal care in response to a CHW intervention. However, the facility improvement work could have improved the quality of care provided during antenatal care visits, intrapartum care and postnatal care. Increasing the utilization of perinatal services with potential improvement in quality of care across perinatal continuum of care may have led to a reduction in maternal and neonatal adverse outcomes. Measuring changes in improvement in quality of ANC, maternal and neonatal outcomes could have strengthened the evidence of the impact our study. However, we did not measure these outcomes in our study.

Interpretation of our study requires viewing the impact of the intervention *as a whole*, including the CHW activities and the facility improvement work. It would have been challenging to implement this program without improving care at facilities and addressing some pertinent community challenges. With Malawi facing a huge burden of both infectious and many chronic non-communicable diseases, the health system is under-resourced, and the need for improving the intervention site to provide emergency obstetric care was identified during the planning phase of the study [3]. Facility support is also mentioned as a pre-requisite for community-based support for perinatal care by the new World Health Organisation guidelines, which advocates improving facility quality of care in community interventions aimed at increasing perinatal service utilisation [27].

### Conclusions and recommendations

Community health workers working with women to provide community education, pregnancy identification, and referral increased the timing and frequency of ANC visits and the proportion of women utilising intrapartum care. Based on the lessons learned from this intervention, the CHW program was re-designed across Neno District and CHWs district-wide were changed from a patient-based distribution to a household-based distribution. Additionally, the package of services provided by CHWs was increased to cover an additional five medical conditions. This new approach, which was rolled out in phases beginning in 2017, will be evaluated in mid-2019 [35]. Additionally, lessons from this study can also be adopted and implemented in other districts in Malawi and other countries who are struggling with low utilisation of perinatal services. Future research should investigate reasons why CHWs did not improve the utilization of postnatal care and also measure the impact of CHWs on neonatal and maternal outcomes in Neno district.

## Supplementary information

**Additional file 1:.** Adjusted population of Neno health facilities in 2015 (projected from 2008 national census).

## Data Availability

The data used in this study was obtained from the Malawi Ministry of Health and as a result the data cannot be made publicly available. However, if the data is needed, please contact the corresponding author of the paper.

## Abbreviations

ANC: Antenatal Care
CHW: Community Health Workers
MDG: Millennium Development Goals
