# Supplementary material for: Increasing utilisation of perinatal services: estimating the impact of community health worker program in Neno, Malawi
Source: BMC Pregnancy Childbirth. 2020 Jan 6;20:22. doi: 10.1186/s12884-019-2714-8 (PMC6945430; doi:10.1186/s12884-019-2714-8)
Supplement: Supplementary file 1 — Additional file 1:. Adjusted population of Neno health facilities in 2015 (projected from 2008 national census). [file 12884_2019_2714_MOESM1_ESM.docx]

**Additional file 1**

**Health facilities in Neno, Malawi in 2015**

| **Facility Name** | **2015 Projected Population** | **2015 Number of Households** |
| --- | --- | --- |
| Chifunga* | 10,900 | 2,180 |
| Ligowe** | 11,345 | 2,269 |
| Lisungwi* | 9,360 | 1,926 |
| Luwani** | 4,567 | 913 |
| Magaleta** | 8,267 | 1,653 |
| Matandani*** | 9,961 | 1,992 |
| Matope*** | 16,739 | 3,348 |
| Midzemba** | 12,913 | 2,583 |
| Neno District Hospital** | 18,927 | 3,785 |
| Neno Parish*** | 6,129 | 1,226 |
| Nkula*** | 2,047 | 409 |
| Nsambe*** | 14,141 | 2,828 |
| Zalewa** | 11,066 | 2,213 |

Table 1: **Adjusted population of Neno health facilities in 2015 (projected from 2008 national census).**

**NB: 1) *Intervention site 2) ** Public facilities used for creating synthetic control 3) *** Facilities with user fees**
